# Predicting the ecological niches of *Aedes aegypti s.l*. using maximum entropy in Kenya

**DOI:** 10.3389/fitd.2025.1641807

**Published:** 2025-10-06

**Authors:** Samuel K. Muchiri, Moses M. Musau, Paul Mwaniki, Fridah Kirimi, Nathan O. Agutu, Emelda A. Okiro, Simon Dellicour, Robert W. Snow

**Affiliations:** 1 Department of Geomatic Engineering and Geospatial Information Systems, Jomo Kenyatta University of Agriculture and Technology, Nairobi, Kenya; 2 Population and Health Impact Surveillance Group, KEMRI-Wellcome Trust Research Programme, Nairobi, Kenya; 3 Spatial Epidemiology Lab (SpELL), Université Libre de Bruxelles, Brussels, Belgium; 4 Institute of Life, Earth, and Environment (ILEE), University of Namur, Namur, Belgium; 5 Department of Public health, Institute of Tropical Medicine Antwerp, Antwerp, Belgium; 6 Data and Statistics, KEMRI-Wellcome Trust Research Programme, Nairobi, Kenya; 7 Centre for Tropical Medicine and Global Health, Nuffield Department of Medicine, University of Oxford, Oxford, United Kingdom; 8 Department of Microbiology, Immunology and Transplantation, Rega Institute, KU Leuven, Leuven, Belgium; 9 Interuniversity Institute of Bioinformatics in Brussels, Université Libre de Bruxelles, Vrije Universiteit Brussel, Brussels, Belgium

**Keywords:** *Aedes aegypti*, ecological niche modelling, maximum entropy, probability of occurrence, arbovirus

## Abstract

**Background:**

To evaluate the human population at risk of arboviral illnesses and improve vector and disease surveillance, it is crucial to model the probability of occurrence of impactful mosquitoes such as *Aedes aegypti sensu lato (s.l.)* which transmits dengue and Chikungunya etc. While majority of studies on *Aedes* distributions have focused on global ecological niche modelling (ENM), there is need to build local vector niche models using national data to design targeted vector surveillance and control strategies. Here, we built a spatial inventory of *Aedes aegypti s.l*. and applied a national-wide ENM approach to predict the probability of occurrence of *Ae. aegypti s.l*. across Kenya.

**Methods:**

Occurrence data on *Aedes aegypti s.l*. from 2000 to 2024 were assembled from the Global Biodiversity Information Facility (GBIF), Walter Reed Biosystematics Unit’s (WRBU) VectorMap, and online literature searches. A maximum entropy approach was used to predict *Ae. aegypti s.l*. probability of occurrence in Kenya for 2024 at ~5 x 5 km resolution, using the occurrence data assembled and environmental covariates: population density, daytime and nighttime land surface temperature (LST), enhanced vegetation index (EVI), elevation, and land cover. Model performance was evaluated using the area under the curve (AUC) metric.

**Results:**

A total of 291 unique locations reported positive identification of *Ae. aegypti s.l*. Population density, daytime and nighttime LST were the most influential predictors. The models predicted high probabilities of occurrence of *Ae. aegypti s.l*. along the coast, northeastern and western Kenya, and in urban centres, while lower probabilities were predicted in sparsely populated areas. The models achieved a mean AUC value of 0.732 (0.653-0.779), indicating a moderate performance.

**Conclusion:**

The predicted distribution of *Ae. aegypti s.l*. can guide vector surveillance in high-risk areas and help identify populations at risk of arboviral diseases like dengue fever and Chikungunya, aiding in future outbreak preparedness.

## 1 Introduction

The mosquitoes of the *Aedes* genera are known to transmit viruses such as dengue virus (DENV), Chikungunya virus (CHIKV), Rift Valley fever virus (RVFV), yellow fever virus (YFV) and Zika virus (ZIKV), and their global expansion with accompanying disease burden is increasing at an alarming rate (1). The main *Aedes* species globally are *Aedes aegypti* and *Ae. albopictus* (2, 3). Dengue fever (DF) has the highest disease burden among human arboviruses, with an estimated 100 to 390 million cases each year and approximately 10,000 deaths globally (4–6). CHIKV has expanded its geographic range to over 100 countries (7) and, as for Rift Valley fever (RVF), it has been reported in over 30 countries in Africa and the Middle East (8). Approximately 339,000 human cases of RVF have been reported in five of nine outbreaks that have occurred between 1997–2010 in Africa (9). Over 100,000 severe infections and over 50,000 deaths due to YFV were estimated in Africa and South America in 2018 (10). ZIKV infected over one million people in over 70 countries during its outbreak in 2015–2016, forcing the World Health Organization (WHO) to declare it a public health emergency of international concern (11). The changing global landscape of *Aedes* transmitted arboviral infections is widely thought to be linked to changing climate, urban settlement patterns, as well as human mobility and trade (12, 13).

In Kenya, the three most significant arboviral infections are DF, Chikungunya (CHIKF) and RVF (14). The first case of DF in Kenya was reported in 1982 in Malindi where the serotype DENV-2 was isolated from a tourist (15). Additional outbreaks of DF have been reported in Mandera in 2011 (16, 17), multiple outbreaks in Mombasa in 2013/2014, 2017, 2019 and 2021 (1, 16, 18–21), Wajir in 2017 (1) and Lamu in 2021 (20). For CHIKF, the first outbreak was reported in 2004 in Lamu, where over 13,000 cases were recorded (22). Other outbreaks have been reported in Mandera in 2016 (23) and Mombasa in 2004, 2018 and 2022 (22, 24). RVFV was first isolated in 1930 from sheep at Lake Naivasha farm (25) where approximately 100,000 sheep died and close to half a million calves aborted (26). In 1997–1998, a major RVF outbreak was reported in East Africa (Kenya, Somalia and Tanzania; 27) and, in 2006–2007, another RVF outbreak was reported in multiple areas across Kenya (27–29). The most recent epidemic occurred in June 2018 in Garissa, Kajiado, Kitui, Marsabit, Tana River and Wajir where the human case fatality ratio was 23% (29).

The increasing outbreaks of *Aedes*-borne arboviral infections in Kenya are a significant public health threat, causing mortality, disability and economic losses (30). The spatial nature of the vector distributions are a fundamental aspect of effective prevention, control and epidemic mitigation strategies. Defining disease vector distributions often employs ecological niche modelling (ENM; 31). ENM approaches are a class of prediction models that combine geo-coded data on locations/areas where the species were present and considered as absent together with the ecological characteristics of those areas to predict the probability of the vectors being observed in space and time given local environmental conditions. These models have previously been applied to the global distribution of *Aedes* species (12, 13, 32–46) and other arthropods including *Anopheles* (47–49) and *Culex* (50) mosquito species, tsetse flies (51), ticks (52), and sand flies (53). However, there are very few examples where these models have been applied for *Aedes* species at national/subnational level in Africa: Kenya (54–57), Morocco (58), Nigeria (59) and Tanzania (60). National/sub-national modelling is important to understand local ecologies using local data to tailor local vector control and surveillance strategies.

The first pillar of WHO’s Global Arbovirus Initiative (GAI) is “monitor risk and anticipate” (61) and emphasizes on a robust surveillance system to integrate historical and current data to map and identify areas at most risk with the aim towards reducing and eradicating *Aedes*-borne diseases. Here, we model the geographic distribution *of Aedes aegypti sensu lato (s.l.)* mosquito species – using the maximum entropy approach implemented in the program MaxEnt – in Kenya, to identify areas that are at high probabilities of *Ae. aegypti* presence that may contribute to arboviral disease outbreaks, inform future vector surveillance priority areas and targeted vector control.

## 2 Methods

### 2.1 Assembling *Aedes aegypti s.l*. occurrence data

Data on *Aedes aegypti s.l*. occurrence in Kenya (Supplementary File 1, Supplementary Figure 1) were extracted from two global inventories: the Global Biodiversity Information Facility (GBIF; http://www.gbif.org) and Walter Reed Biosystematics Unit’s (WRBU) VectorMap (http://www.vectormap.si.edu) and supplemented by an online literature search. The global inventories were accessed on the 14^th^ October 2024. The data attributes of the occurrence data extracted included the location name, county, coordinates (latitude and longitude), year of collection, whether larvae or adults were sampled, the *Aedes* species identified and the data sources. Data that had a combination of missing location name and missing coordinates, records with missing species information and records reporting other *Aedes* species were excluded. Only data from field surveys undertaken from 2000 were included to match available yearly covariate extractions.

The online literature were searched in online databases including EBSCOhost (MEDLINE, Academic Science Complete and CINAHL Complete), Ovid (Embase and MEDLINE), ProQuest Dissertations and Theses Global, PubMed, Scopus, Web of Science, and Google Scholar. The searches were restricted to articles published from 2000 through to 2024. Boolean terms (OR and AND) and truncations (*) were used to improve the search. The key terms used in the database search include “*Aedes*” OR “*Stegomyia*” OR “Mosquito*” AND “Kenya”. The search results were then imported to Zotero (version 7.0.15) for de-duplication and data synthesis. Data extraction was undertaken and entered in Microsoft Excel, including (i) details on the geographical location of each sampling site, including Global Positioning System (GPS) recordings, village and county names, (ii) survey dates and duration of surveillance, (iii) *Aedes* species identified at each sampling site, (iv) whether larvae or adults and (v) publication source citations and sources. These were then merged with equivalent data fields from the GBIF and WRBU-VectorMap data repositories.

The literature searches identified multiple publications reporting on the same surveys and were collapsed to a single row of information per site-time indicating multiple sources. Additionally, the same or different investigators reported the same site sampled at different, but almost contiguous times (within a year) of each other across several publications, these were also collapsed to a single site entry, and surveillance periods were extended. Data from the same sites collected more than 12 months apart were included as separate entries and indicated as spatial and temporal duplicates within the database. The two open-access global repositories, GBIF and WRBU-VectorMap contained information shared across databases and those identified during the literature search. Care was taken to removed likely duplicates or replications of the same survey report.

The geographic information provided in publications and reports were of varying precision and spatial resolutions, sometimes with accompanying maps, more often without. Where information was reported by county, sub-county or broad area only, these were excluded from the database as they extended beyond a ~5 x 5 km resolution grid. Other source data were geo-coded to provide a longitude and latitude for each survey location. Among the data from the GBIF and WRBU-VectorMap repositories not identified in the literature search, we have presumed the locations were provided with Global Positioning System (GPS) coordinates at trap or larval sampling sites. Where these covered multiple sites within a single village, these were combined to the village/year of sampling. All other data were geo-coded using a variety of gazetteer and digital online resources. Decimal degrees longitude and latitude were as a default attributed using GPS coordinates when provided in the report, the village name was searched using descriptions in the report using Google Earth Pro (version 7.3.6.9796) and finally using a national gazetteer of census village names (62). After the geocoding process was completed for all combined data sources, the current county and sub-county names were added from shapefiles sourced from the County Integrated Development Plans (CIDP; https://www.devolution.go.ke/) using the “*Join attributed by location*” tool in Quantum Geographic Information System (QGIS) software (version 3.32.2).

### 2.2 Covariates selection

**Figure 1:**
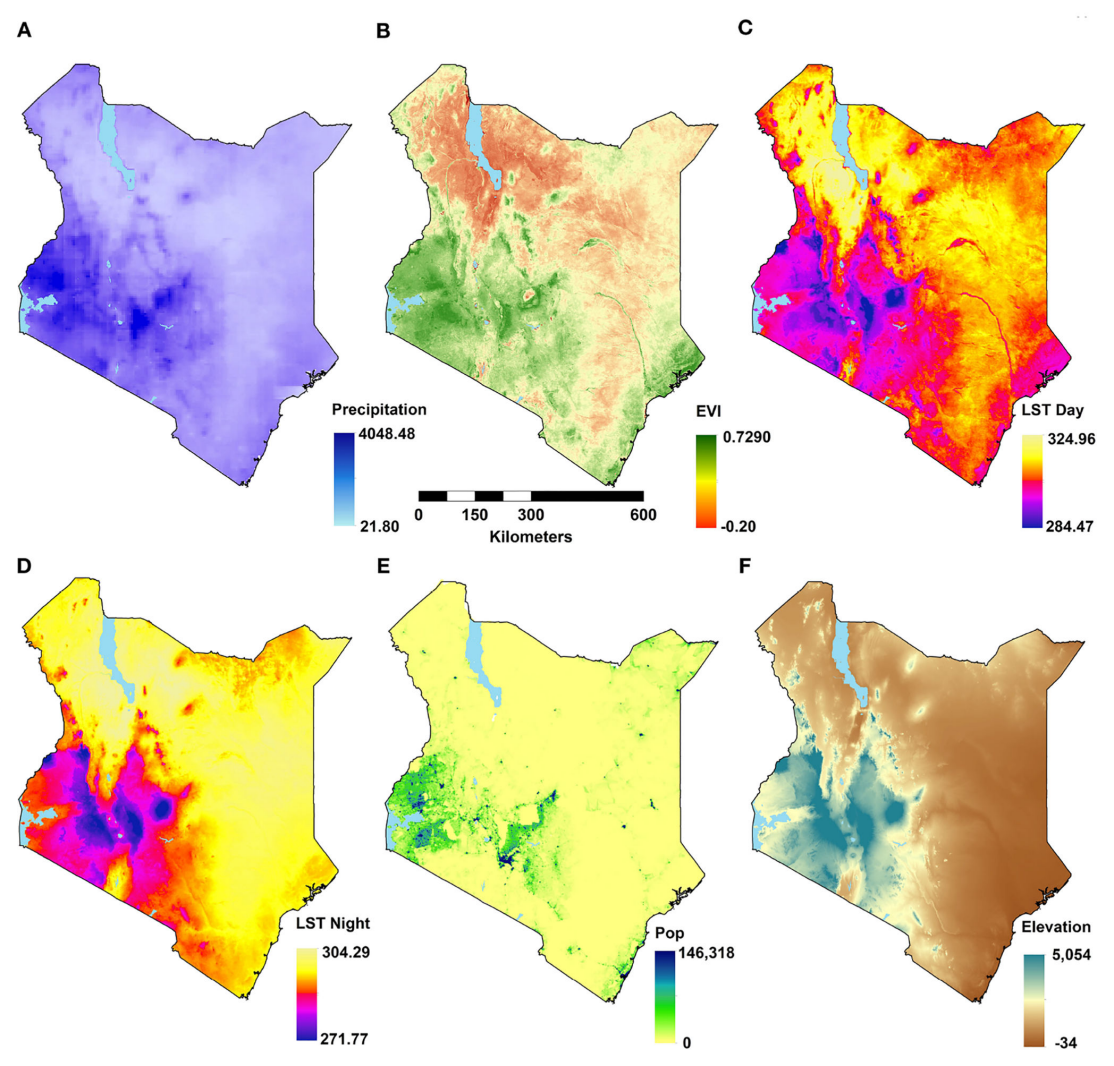
Maps of environmental covariates at their original resolutions for the latest years (see Table 1) showing: annual total rainfall **(A)**, annual mean enhanced vegetation index (EVI; **B**), annual daytime land surface temperature (LST; **C**); annual nighttime LST **(D)**; population density **(E)**, and elevation **(F)**.

**Figure 2:**
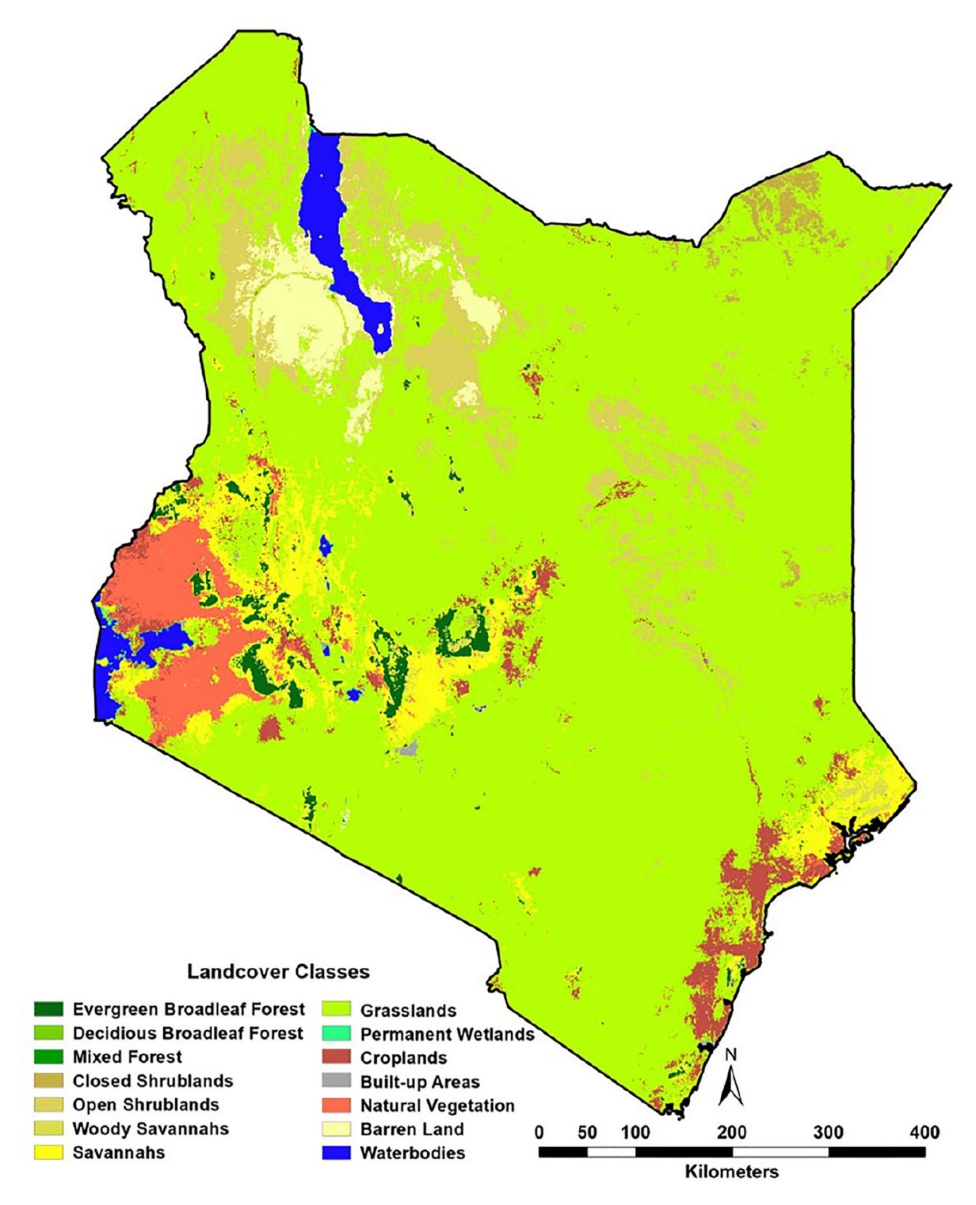
Moderate Resolution Imaging Spectroradiometer (MODIS)-defined land cover for 2023.

A suite of potential covariates to be integrated in the ENM analyses were selected from a systematic review undertaken for *Aedes* ecological niche modelling (63). Across 113 studies reviewed, the frequently used covariates for *Aedes* species modelling included rainfall, temperature, elevation/slope, vegetation indices, population density and land cover (Table 1; Figures 1, 2; 63). More information on their relationship with suitability of *Ae. aegypti* has been detailed in Supplementary File 1. Rainfall, daytime and nighttime land surface temperature (LST), elevation, enhanced vegetation index (EVI) and land cover datasets were accessed and downloaded through the Google Earth Engine (GEE) platform (https://earthengine.google.com/). Population density data was accessed from the WorldPop website (https://www.worldpop.org/). We generated distinct binary rasters for each land cover class to be considered independently in the ENM analyses where a cell value of 0 indicated the absence of the considered land cover variable and 1 its occurrence. For the analysis presented in this manuscript, the covariates were then resampled to a spatial resolution of approximately 5 x 5 km and were linked and extracted to the presence locations for the relevant start year of collection.

For the models to produce accurate and reliable results, the covariates were tested for multicollinearity using Pearson’s correlation coefficient with a cut-off value of |0.7|. The covariates’ relative importance (RI) were then tested using the Jackknifing procedure within MaxEnt (64). RI is a percentage measure of how much the model performance drops by when the variables are randomly permuted. For highly correlated variables (>|0.7|), the covariate with the higher importance was maintained for prediction leaving a set of relatively uncorrelated variables.

### 2.3 Maximum Entropy (MaxEnt) analyses

Maximum entropy (MaxEnt) is a general-purpose ENM approach for making predictions and widely used in ENM analyses conducted for *Aedes* spp. distribution modelling (63). MaxEnt aims to estimate a target probability distribution by finding the probability distribution of maximum entropy (i.e. most spread out or closest to uniform; 64), subject to a set of constraints/rules that represent our incomplete information about the target distribution. The target/unknown probability distribution, which is denoted as *π* is over a finite set *X* (set of pixels). The distribution *π* assigns a non-negative probability *π*(*x*) to each point (*x*; 64). The approximation of *π* is also a probability distribution denoted as *ω*.

The entropy is defined as:

H(ω)=⊢∑ω(x)ln(ω(x))

Where *ln* is the natural logarithm. Entropy as defined by Shannon is a measure of how much ‘choice’ is involved in the selection of an events. Thus, a distribution with higher entropy involves more choices (less constrained; 65). MaxEnt provides a logistic output that considers the species’ prevalence, which is defined as the fraction of occupied places. MaxEnt’s default prevalence value of 0.5 indicates that the species is present in half of all feasible sites.

MaxEnt software (version 3.4.4) was used for implementation of the MaxEnt model predictions (66). All MaxEnt features (linear, quadratic, hinge, produce, and threshold) were tested, and the appropriate regularization parameters (for penalising complexity) were evaluated using the Corrected Akaike Information Criterion (AICc) using the “ENMeval” R package. The features/constraints with the lowest AICc value were used in the final model. Presence data is often clustered around areas of known epidemics. Due to lack of presence records in some locations and absence data identified in the *Ae. aegypti s.l*. data assembled, we created a bias file using a kernel density estimation (KDE) surface created from the presence points. We generated the same number of pseudoabsence points as the presence points, and those pseudo-absence points were solely simulated within the areas of highest density of presence points to match with the sampling bias of presence points. We ran 10 MaxEnt replicate analyses, and the average prediction and standard deviation surfaces were produced.

### 2.4 Model evaluation

Presence and pseudo-absence points were split randomly into 10 folds for MaxEnt replicate analyses using the k-fold cross-validation technique and the area under the receiver operating characteristic curve (AUC) statistic calculated on the validation sets. AUC is a measure of how well the model differentiates suitable and non-suitable areas and ranges from 0 to 1 where a value of 0.5 indicates a random model (67), and values above 0.7 indicating reliable estimates (68). We report the final average AUC, its 95% confidence intervals (CI) and the AUC plot of sensitivity against 1 – specificity. Sensitivity is defined as the probability of a true positive while specificity is the probability of a true negative result. Essentially then, 1 – specificity returns the false positive rate. K-fold cross-validation and calculation of the AUC values were conducted with the program MaxEnt.

## 3 Results

### 3.1 Occurrence data assembled

524 records were extracted from the GBIF repository. 136 were excluded as no information existed on the survey location and 284 pre-2000 surveys were excluded (Supplementary File 1, Supplementary Figure 2). The remaining 388 GBIF site location data were collapsed to single site entries as they were spatial and temporal duplicates. For spatial duplicates covering multiple years of collection, these were separated to annual periods, and there was a record for each spatial duplicate for each year. The final GBIF site location data covered 30 time-site locations between 2007 and 2024. 3,388 records were extracted from the WRBU VectorMap database. Through a process of de-duplication and collapsing to single time-sites only, 85 time-site records remained sampled between 2005 and 2021 (Supplementary File 1, Supplementary Figure 2).

After the literature review and de-duplication, 266 records at 231 time-site locations were eventually extracted. It was possible to link 19/30 site specific entries extracted from the GBIF database to the publications identified during the online literature searches. Similarly, data extracted from the WRBU VectorMap portal were cross-referenced to the data sourced from the online literature searches. 26/85 site entries in the WRBU-VectorMap data were identified during the online literature searches (Supplementary File 1, Supplementary Figure 2). 221 occurrence records were identified from online literature sites which were not in both GBIF and WRBU VectorMap databases and they were combined into a single database.

**Figure 3:**
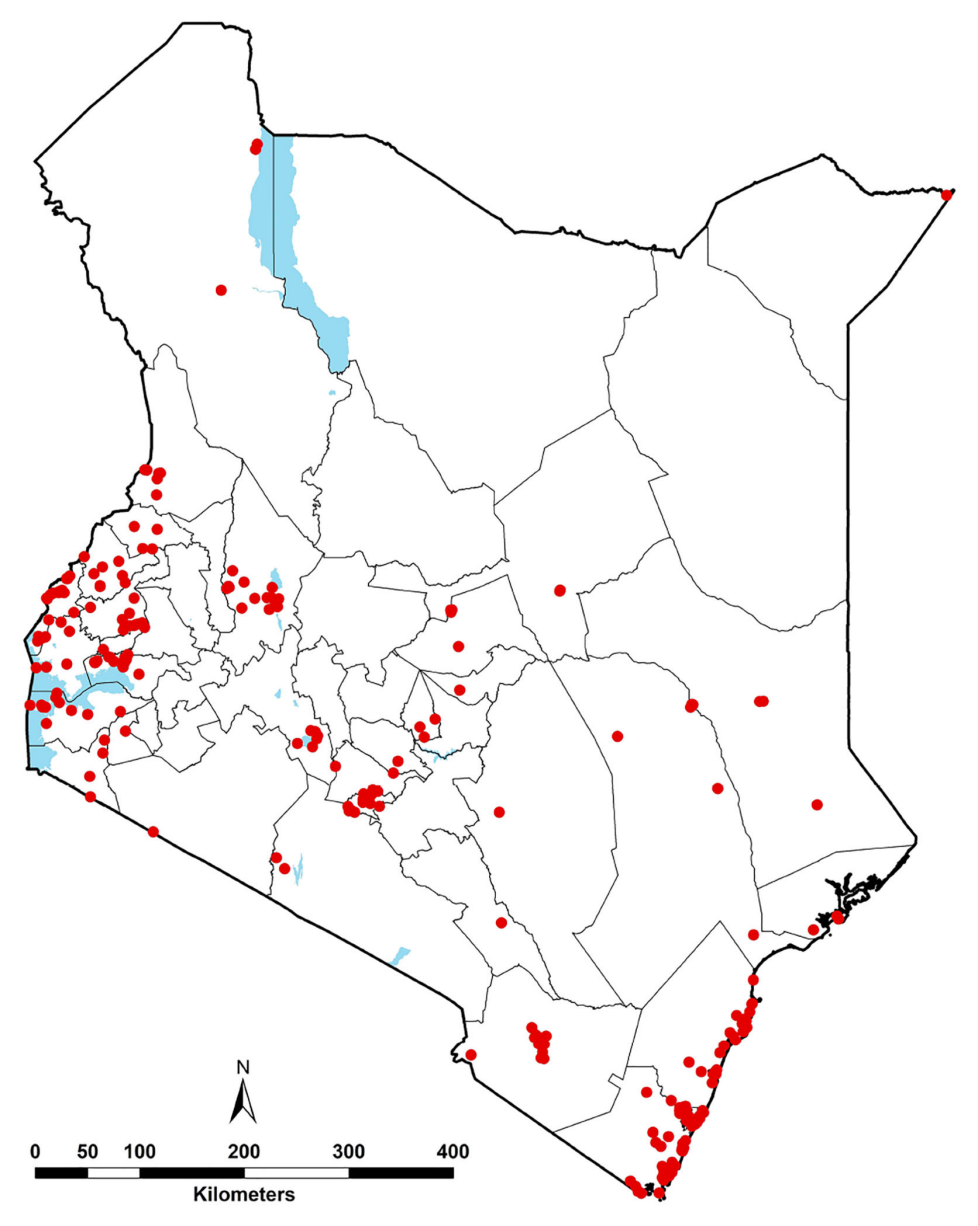
Geographic distribution of *Ae. aegypti s.l*. occurrence records between 2000 and 2024 (red dots), i.e. 328 unique records corresponding 291 unique locations. The waterbodies are displayed in blue and county boundaries in grey.

The final composite database provided information of *Ae. aegypti s.l*. from 336 records. However, 8 records corresponded to administrative polygons and were excluded from the analyses, which led to a total of 328 occurrence records at 291 unique locations within a ~5 x 5 km grid sampled between 2000 and 2024 (Supplementary File 1, Supplementary Figure 2). The geographical distribution of the combined wide-area and point data between 2000 and 2024 where *Ae. aegypti s.l*. had been sampled is shown in Figure 3.

### 3.2 Covariate selection

**Figure 4:**
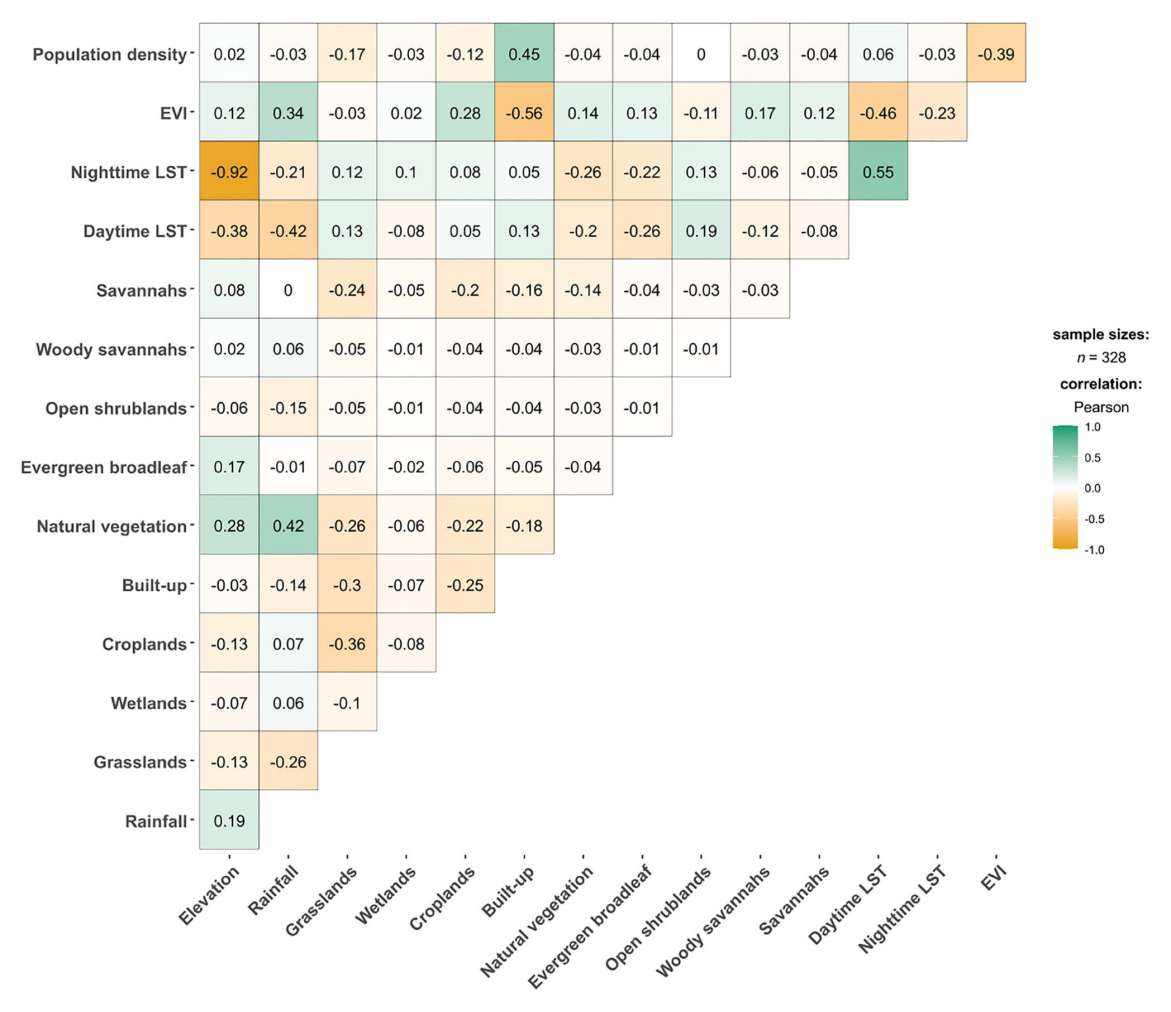
Pearson’s correlation coefficient matrix results for environmental covariate values extracted from *Aedes aegypti s.l*. occurrence points. “LST” refers to land surface temperature; “EVI” refers to “enhanced vegetation index”.

Environmental covariates were extracted at *Ae. aegypti s.l*. presence locations prior to a preliminary correlation analysis. Elevation and nighttime LST showed a strong negative correlation (*ρ* = -0.92). Elevation was excluded from the model due to its lower RI (1.2%) compared to 3.2% RI by nighttime LST from the Jackknifing method. Built-up areas and population density — both indicators of urbanisation — were moderately correlated (*ρ* = 0.45; Figure 4). Other covariates exhibited low correlations and were retained for the ENM analyses.

### 3.3 Prior analysis to select the optimal settings for final model

We tested a combination of different MaxEnt features (linear, quadratic, product, threshold and hinge) and different regularisation multipliers using the “ENMevaluate” R package to select the optimal settings to be applied to the *Ae. aegypti s.l*. final ecological niche models. The model with the combination of linear, quadratic and hinge features with the regularisation multiplier of one was selected as the best model as it had the lowest AICc (3237.52) followed closely by a model with hinge features only and a regularisation multiplier of one (AICc = 3245.99). The combination of linear and quadratic features with a regularisation parameter of five was the worst model (AICc = 3423. 90; Supplementary File 1, Supplementary Figure 3).

### 3.4 Model prediction performance

**Figure 5:**
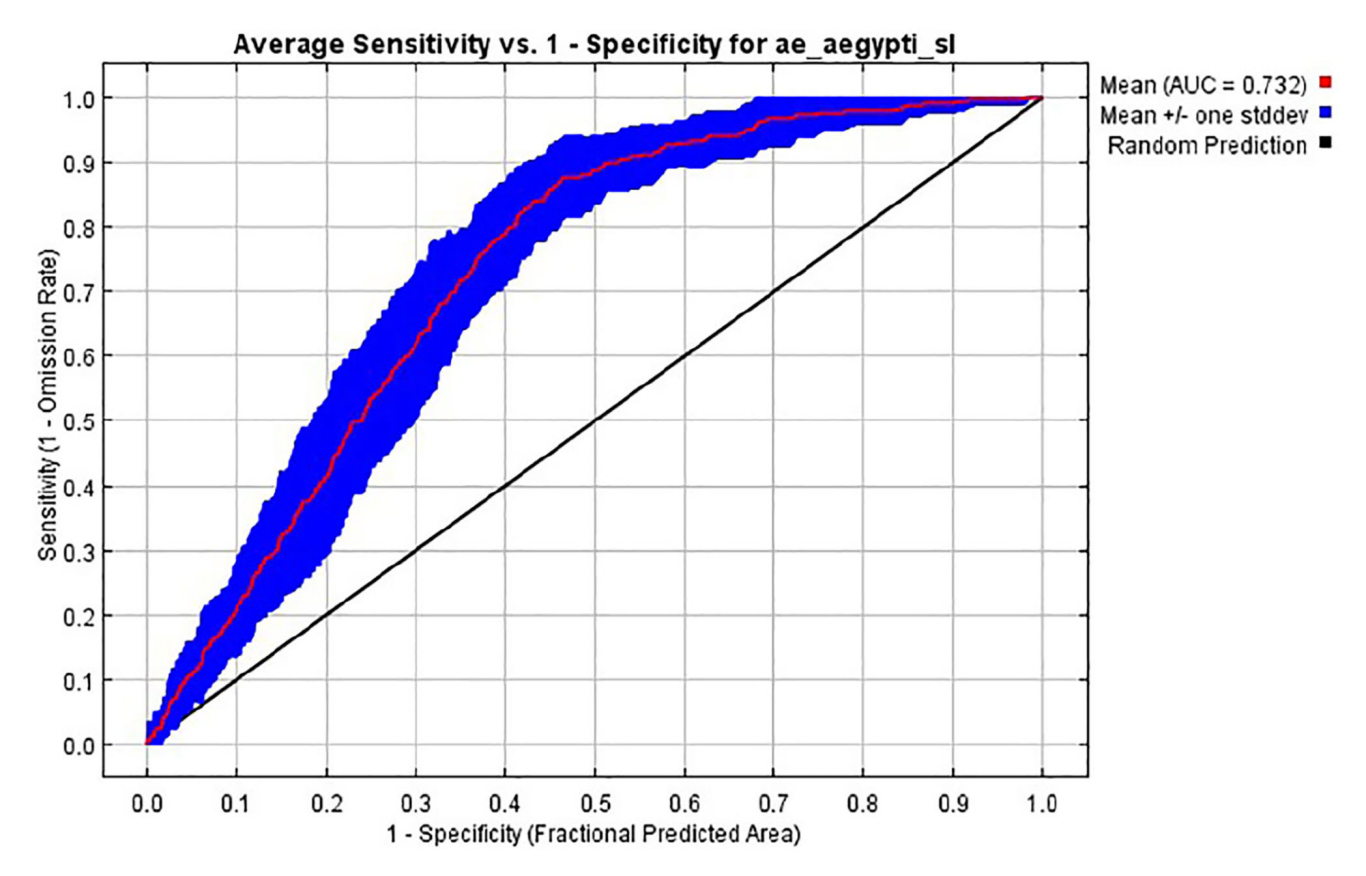
The receiver operator characteristic (ROC) curves used to estimate the area under the curve (AUC) measuring the predictive of the ecological niche models trained for *Ae. aegypti s.l*., and here averaged over ten replicate analyses (red curve). The blue area shows the area of uncertainty (+/-standard deviation).

The predictive performance of the ecological niche models trained for *Aedes aegypti s.l*. species was evaluated using the area under the receiver operating characteristic curve (AUC; Figure 5). The AUC curve plots sensitivity (1 - omission rate/true positive rate) against 1 - specificity (false positivity rate). The mean AUC curve, represented by the red line, achieved AUC of 0.732 (0.653-0.779), indicating a moderate prediction model (Figure 5). The blue shaded area around the mean curve represents one standard deviation, measuring variability in the model’s performance. The black diagonal line represents a random prediction baseline. The high AUC value demonstrates the model’s effectiveness in distinguishing between suitable and unsuitable habitats for *Aedes aegypti s.l*.

### 3.5 Modelled probability of occurrence of *Aedes aegypti s.l*.

**Figure 6:**
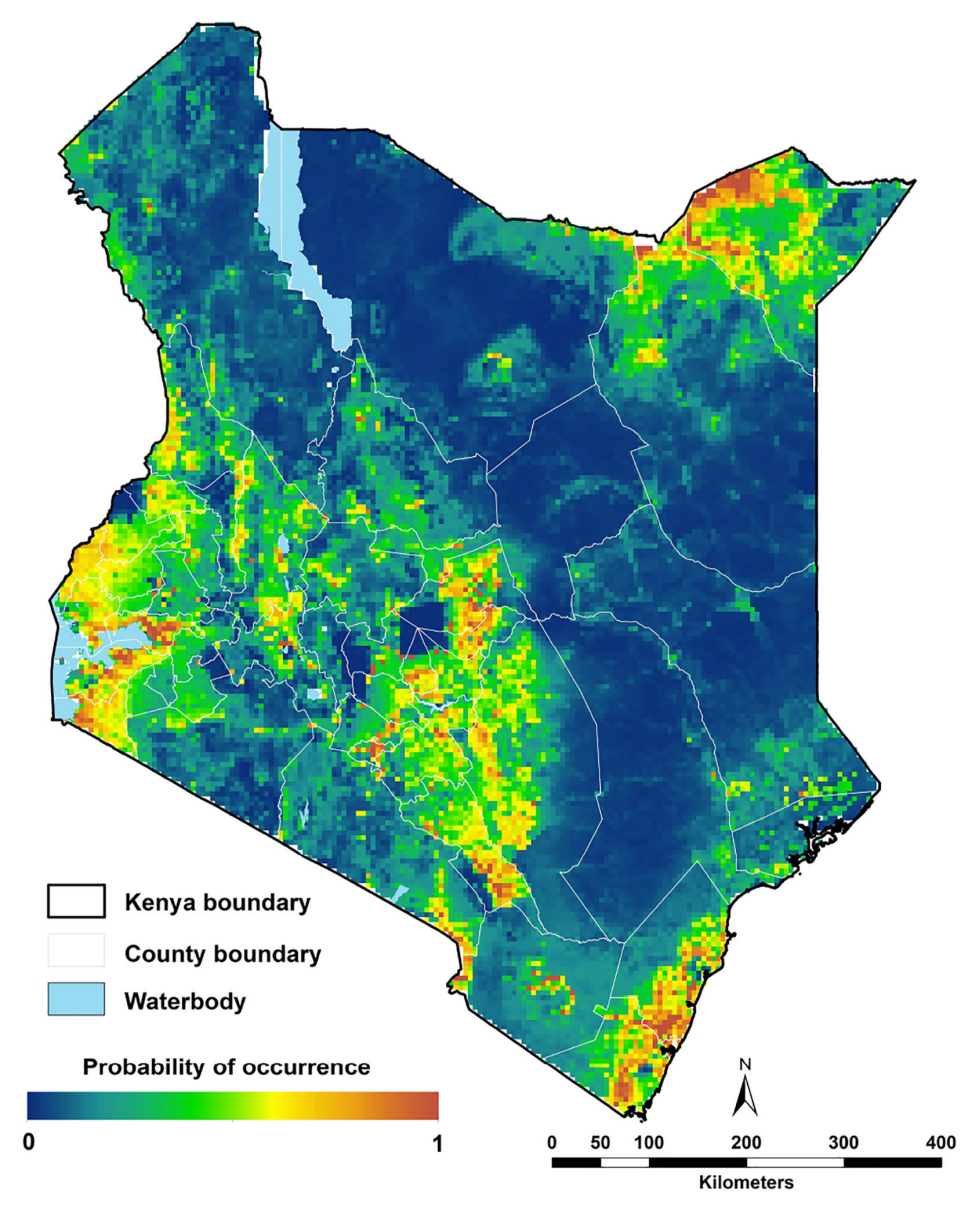
Ecological niche modelling analysis for *Aedes aegypti s.l*. in 2024 in Kenya. High probabilities of occurrence were predicted along the coast, the northeastern central and western parts and low probabilities of occurrence predicted in sparsely populated areas (Figure 1E).

The highest probabilities of occurrence of *Ae. aegypti s.l*. were predicted along the coast, in western Kenya around Lake Victoria and areas in northeastern Kenya in Mandera and Wajir (Figure 6). More specifically, the highest probabilities were estimated around urban centres, with prediction probabilities ranging between 0.6 and 1. The lowest probabilities of occurrence (between 0.0 and 0.2) were estimated for the high elevation areas including Mount Kenya, Mt. Elgon, the Aberdare Forest and Mau escarpments, Mt. Marsabit, across forested areas and in areas where the population is less than 1,000 people per ~5 x 5 km grid cell including protected areas (national parks and reserves; Figure 6; Supplementary File 1, Supplementary Figure 1). The prediction uncertainties (standard deviation) ranged from 0 to 0.14 with the highest uncertainty values in the northeastern part of Kenya in Marsabit, Isiolo, Wajir and Mandera and the lowest values around in the protected areas in the Maasai Mara and Tsavo national parks and the high elevation areas where the lowest probabilities were predicted (Supplementary File 1, Supplementary Figures 1, 4).

### 3.6 Covariate relative importance

Population density was the most important variable (52.7%), followed by daytime LST (40.5%) and nighttime LST (3.2%). Evergreen broadleaf land cover variable followed with a 1.9% RI. Woody savannah land cover variable had a 0.8% RI while rainfall had a 0.6% RI. Permanent wetlands and savannahs had a 0.1% RI each. Barren areas, built-up, closed shrublands, croplands, deciduous broadleaf, EVI, grasslands, mixed forest, natural vegetation, open shrublands, and water had zero RI to the model (Table 2).

### 3.7 Response curves

The response curves illustrate the individual effects of each environmental covariate on predicted probabilities of occurrence/habitat suitability, thus aiding in the interpretation of the ecological preferences of a given species. For population density, there is a sharp increase in the predicted probability of occurrence with an increase in population density and maximum probability is reached when the population density is 1,000 people per ~5 x 5 km grid cell, and from there it plateaus, showing there is no difference in increase in population density and the probability of occurrence of *Ae. aegypti s.l*. (Supplementary File 1, Supplementary Figure 5).

For daytime LST, the predicted probability of occurrence increases with an increase in temperature up to 303 K (29.9°C) where the predicted probability of occurrence is 0.72 (Supplementary File 1, Supplementary Figure 5). There is a very slight reduction in probabilities from 0.72 up to ~0.70 at 313 K (39.9°C) where the probability of occurrence drops slightly again to 0.68 at 314 K (40.9°C) from which the probability remains at 0.68 until the maximum daytime LST (~316 K; 42.9°C). Similarly, the probability of occurrence of *Ae. aegypti s.l*. increased exponentially with an increase in nighttime LST up to 288 K (14.9°C) where the probability of occurrence is 0.62. There is a linear increase in probability of occurrence between 288 K (14.9°C) and 297 K (23.9°C) where the probability then increases to 0.72. The probability of occurrence then starts to fall until maximum nighttime LST of 304 K (30.9°C) where the probability of occurrence is close to 0 (Supplementary File 1, Supplementary Figure 5).

For rainfall, there is then a very sharp increase in probability from 0.18 at 200 mm of annual rainfall to 0.65 at ~700 mm of annual rainfall. The probability of occurrence of *Ae. aegypti s.l*. then reduces exponentially from 0.65 at ~700 mm of total annual rainfall to 0.46 at ~4000 mm of annual rainfall thereafter a plateau in probability until maximum rainfall (Supplementary File 1, Supplementary Figure 5).

The probability of occurrence of *Ae. aegypti s.l*. was lower in evergreen broadleaf areas (0.44) than in non-evergreen broadleaf areas (0.63). There was an almost equal probability of occurrence of *Ae. aegypti* in areas classified as woody (0.62) and non-woody savannah areas (0.63). In contrast, the predicted probability of occurrence was higher in permanent wetland areas than in non-permanent wetland areas (0.71 vs 0.63 respectively). For areas covered by savannah, the predicted probability of occurrence was lower (0.6) than in non-savannah areas (0.64; Supplementary File 1, Supplementary Figure 6).

## 4 Discussion

The study assembled data on the occurrence of *Aedes aegypti s.l*. species in Kenya at 291 unique locations from 2000 to 2024 and predicted the probability of occurrence using the ecological niche modelling approach implemented in the program MaxEnt. Using the maximum entropy approach available in this program, we identified population density, daytime and nighttime LST as the most influential predictors of *Ae. aegypti s.l*. probability of occurrence and the model had a moderate predictive performance (AUC = 0.732; 0.653-0.779). The highest probabilities of occurrence for *Ae. aegypti s.l*. were predicted along the coast and northeastern Kenya ranging between 60% and 100% occurrence probabilities, and the lowest probabilities in high altitude and low population density areas of the country.

High prediction *Aedes aegypti s.l*. probabilities from the model include the northeastern, coastal and western areas of the country (Figure 6). It is worth mentioning that the current study predicts high probabilities in some areas in the northeastern region where no occurrence data were available (Figure 3), however the current environmental conditions are suitable. In the past ten years, outbreaks of DENV and CHIKV have been documented in the northeastern (1, 16, 17, 23) and coastal Kenya (16, 18, 20, 24), which is consistent with the high probabilities predicted by our ENM analyses. Furthermore, evidence from existing literature report high seroprevalences of DENV and CHIKV along the Kenyan coast corroborating with our findings. The standard deviation of the model probabilities were moderate to high in northeastern Kenya (Supplementary File 1, Supplementary Figure 4). The value of the variance lies in guiding future surveillance efforts to prioritise areas with high prediction uncertainties and/or sparse data coverage. It is important to note that despite high probabilities of occurrence western Kenya, no arboviral disease outbreaks have been reported there, although seroprevalence studies indicate moderate to high exposure to DENV and CHIKV (69–71).

The probability of occurrence of *Ae. aegypti s.l*. in Kenya was mainly driven by population density (Table 2; Figure 1E). Population density has been identified as the most important variable for *Ae. aegypti* in multiple studies (54, 72–75). *Aedes aegypti* has been described to be involved in the urban transmission cycle of various arboviruses and this confirms the species as an urban vector (76). Four studies have identified urban variables i.e. urban building density, percent urban, built-up areas as a categorical variable (contrary to the study’s result) and distance to urban areas as the most important variables (77–80).

Daytime and nighttime LSTs have also been identified as important covariates determining the presence of *Ae. aegypti s.l*. (Table 2; Figures 1C, D). From the ENM analyses, *Ae. aegypti s.l*. was found to be highly suitable in areas where the daytime LST is between 29.9°C and 42.9°C. For nighttime LST, *Ae. aegypti s.l*. probabilities rose between ~2°C and 23.9°C (Supplementary File 1, Supplementary Figure 5). Brady et al. (81) estimated that the optimal temperatures for *Ae. aegypti* survival was about 21°C but the species can survive temperatures from 0°C to 40°C though for both extremes, they survive for a very short time.

Surprisingly, rainfall was one of the least important variables for *Ae. aegypti s.l*. suitability in predicting occurrence (Table 2; Figure 1A). Two studies have predicted precipitation of the driest month/quarter as the most important variable for the probability of occurrence of *Ae. aegypti s.l*., in Tanzania (60) and the other in China (82). However, many studies have also shown that other factors such as temperature, population, land cover and dry season duration have a more dominant impact on *Ae. aegypti* presence than rainfall (37, 42, 83–87). In concert with rainfall, EVI had zero RI (Table 2; Figure 1B). This was contrary to global ecological niche modelling studies of *Ae. aegypti s.l*. where EVI was a dominant variable for model predictions (12% RI; 42) and (8% RI; 37).

It is notable for example that the widely cited global *Ae. Aegypti* and *Ae. albopictus* predictions show almost universal high probabilities of occurrence of both species across Kenya’s landmass (42). Using global models, and globally defined covariate associations with restricted occurrence data per country (in Kenya only 42 presence locations were included – all *Ae. aegypti*; 42) might reduce prediction accuracies at country-level scales. *Ae. albopictus* has yet to be identified in Kenya (42, 88). However, with the *Ae. albopictus* rapid niche expansion due to factors such as increased urbanicity and human mobility, the species is a threat to future arboviral epidemics in the country (88). There is need to build national level geo-coded inventories and nationally defined ecological niche modelling to support national level vector control and disease predictions.

The present study is associated with a series of limitations that might be addressed in future work. The maximum entropy approach used in the current analysis led to ecological niche models associated with a relatively high predictive performance, but there are several caveats. First, this approach adopts an exponential model for probabilities, which is unbounded and may yield inflated predictions for environmental conditions beyond the study area’s observed range (89). Second, MaxEnt is somehow a black box, i.e. it relies on an assumption of prevalence and hence cannot perform your own spatial cross-validation and predicts many false absences (90, 91). Despite a high predictive performance, some assumptions apply, including interspecific competition between species was not considered and nor was dispersal/movement of species. However, the results of the model are a starting point to prioritize surveillance in highly probable areas. Furthermore, other covariates were not explored including access to roads, rivers and waterbodies, livestock density, within urban agricultural areas and other land use surfaces. Future work should explore these local covariates at higher resolution for improved predictions.

We have used two open access geo-coded databases to identify reported sampling of *Aedes aegypti s.l*. in Kenya. However, it is important to note that a formal literature search identified an additional 221 records not found in these data repositories (Supplementary File 1, Supplementary Figure 2). This highlights the need to constantly update mosquito species inventories to provide nationally led data platforms for future distribution modelling.

Most arboviral diseases including DF and CHIKF, are classified as neglected tropical diseases (NTDs) and are often absent from the global health agenda, resulting in limited resources and access to global funding support (92). For instance, very little is known about the epidemiology of CHIKV in Africa and in Kenya (93, 94). Most vector surveillances in Kenya are focused on *Anopheles* species given the persistent endemicity of malaria. However, we must also consider the potential emerging threats posed by arboviral diseases that are transmitted by these less-studied vectors and future vector surveillance should be encouraged to include *Aedes* and *Culex* species sampling.

## 5 Conclusion

The goal of the study was to use occurrence data together with publicly available environmental covariates to predict the probability of occurrence *Ae. aegypti s.l*. in Kenya given local environmental condition. This research serves as a foundation for future studies to compare various ecological niche modelling approaches. The modelling results are vital for identifying areas at risk of arboviral infections such as dengue fever and Chikungunya, predicting potential establishment zones, and enhancing vector surveillance in regions with poor entomological reporting, ultimately aiding in vector control efforts.

## Supplementary Material

The Supplementary Material for this article can be found online at: https://www.frontiersin.org/articles/10.3389/fitd.2025.1641807/full#supplementary-material

SI 1

SI 2

## Figures and Tables

**Table 1:** Covariates used and their key characteristics (see Supplementary Figures 2, 3 for the panel of covariates for Kenya).

Covariate	Source	Type	Units	Interval	Temporal extents	Spatial resolution
Rainfall	CHIRPS	Dynamic	Total Rainfall in mm	Annual	1981-Date	0.05 decimal degrees (~5 x 5 km)
Enhanced Vegetation Index (EVI)	MODIS	Dynamic	–	Annual	2000-Date	~250 x 250 m
Land Surface Temperature (LST) – Day and Night	MODIS	Dynamic	Kelvin	Annual	2000–Date	~1 x 1 km
Elevation	SRTM	Static	Metres	–	2000	~30 x 30 m
Land cover	MODIS	Dynamic	Land cover classes	Annual	2001-2023	~500 x 500 m
Population density	WorldPop	Dynamic	Population per pixel	Annual	2000-2024	~1 x 1 km

**Table 2:** Summary of covariate relative importance in the prediction models for *Aedes aegypti s.l*. “LST” refers to land surface temperature.

Environmental covariates	Relative importance to the ecological niche model
Population density	52.7% (48.0-57.3)
Daytime LST	40.5% (36.3-43.6)
Nighttime LST	3.2% (2.4-5.0)
Evergreen broadleaf	1.9% (0.8-2.8)
Woody savannahs	0.8% (0.3-1.4)
Rainfall	0.6% (0.3-0.9)
Permanent wetlands	0.1% (0-0.3)
Savannahs	0.1% (0-0.3)
Barren	0%
Built-up	0%
Closed shrublands	0%
Croplands	0%
Deciduous broadleaf	0%
Enhanced vegetation index (EVI)	0%
Grasslands	0%
Mixed forest	0%
Natural vegetation	0%
Open shrublands	0%
Water	0%

The values in brackets indicate their 95% credible intervals.

## Data Availability

The data analyzed in this study is subject to the following licenses/restrictions: The occurrence data is subject to further updates and it will be readily available with its own publication. Requests to access these datasets should be directed to smuchiri@kemri-wellcome.org or rsnow@kemri-wellcome.org.
