# Supplementary material for: Predicting the ecological niches of Aedes aegypti s.l. using maximum entropy in Kenya
Source: Front Trop Dis. Author manuscript; Available in PMC 2026 Jan 20. (PMC7618658; doi:10.3389/fitd.2025.1641807)
Supplement: SI 1 [file EMS212068-supplement-SI_1.pdf]

Supplementary material

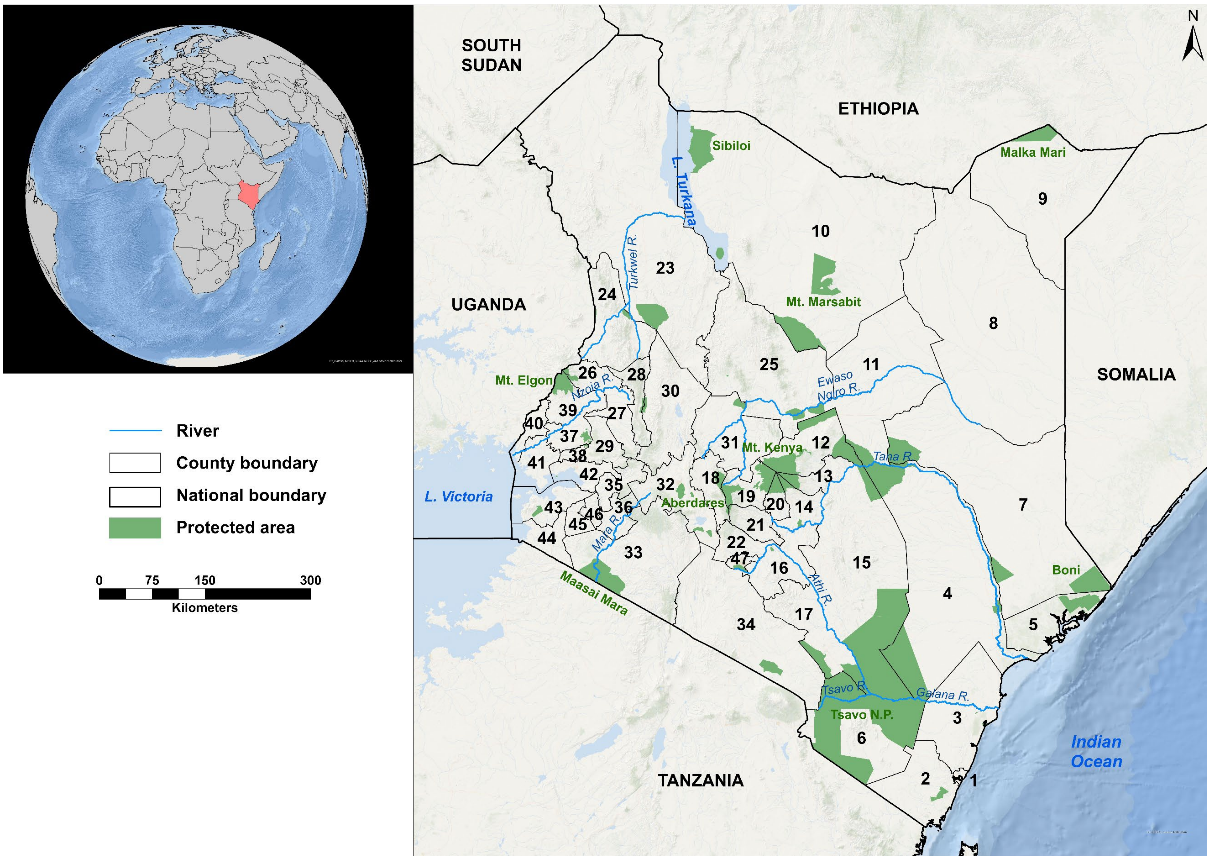

Figure SI 1: Map of Kenya with the 47 counties: Mombasa **[1]**, Kwale **[2]**, Kilifi **[3]**, Tana River **[4]**, Lamu **[5]**, Taita Taveta **[6]**, Garissa **[7]**, Wajir **[8]**, Mandera **[9]**, Marsabit **[10]**, Isiolo **[11]**, Meru **[12]**, Tharaka-Nithi **[13]**, Embu **[14]**, Kitui **[15]**, Machakos **[16]**, Makueni **[17]**, Nyandarua **[18]**, Nyeri **[19]**, Kirinyaga **[20]**, Murang'a **[21]**, Kiambu **[22]**, Turkana **[23]**, West Pokot **[24]**, Samburu **[25]**, Trans Nzoia **[26]**, Uasin Gishu **[27]**, Elgeyo-Marakwet **[28]**, Nandi **[29]**, Baringo **[30]**, Laikipia **[31]**, Nakuru **[32]**, Narok **[33]**, Kajiado **[34]**, Kericho **[35]**, Bomet **[36]**, Kakamega **[37]**, Vihiga **[38]**, Bungoma **[39]**, Busia **[40]**, Siaya **[41]**, Kisumu **[42]**, Homa Bay **[43]**, Migori **[44]**, Kisii **[45]**, Nyamira **[46]**, Nairobi **[47]**.

Covariates used

The principal larval habitats of *Aedes* species especially urban Aedes species such as *Ae. aegypti* and *Ae. albopictus* are man-made containers that are used for water storage and this water may be collected from accumulated rain (1,2). Studies have shown a high vector abundance in areas of high rainfall (3,4). Rainfall is also an indicator of increased socio-economic activities including cultivating, working and grazing in bushes where *Aedes* mosquitoes rest during the day thus increasing the risk of vector-human contacts and virus infection (5). Rainfall data was obtained from Climate Hazards Group InfraRed Precipitation with Station (CHIRPS). The dataset was created by incorporating satellite imagery and in-situ data globally to create a gridded surface of total rainfall (6–8). CHIRPS ranges from 1981 to date at a spatial resolution of 0.05 x 0.05 decimal degrees (approximately 5 x 5 km).

Aedes species are greatly affected by temperature, and this influences larval development and adult survival. Temperatures below the optimum range limit the development of larvae and

temperatures above the optimum reduce the survival of these vectors at any life stage (9). Land surface temperature (LST) at daytime and nighttime were used in this study, and they were obtained from the Moderate Resolution Imaging Spectroradiometer (MODIS) sensor. The dataset ranges from 2000-date at a spatial resolution of approximately 1 x 1 km.

Elevation is used as a proxy for temperature but may also have an independent effect on ecologies related to highlands and mountains. Elevation was obtained from the 30 x 30 m Shuttle Radar Topography Mission (SRTM), and this was also used to calculate the slope in degrees in ArcMap version 10.8 (ESRI Inc., Redlands, CA, USA). The dataset ranges from 2000-date.

Enhanced vegetation index (EVI) measures vegetation canopy greenness and can be used as a proxy for soil surface-level moisture that is associated with the availability of breeding environments (10,11). EVI was derived from MODIS sensor imagery at a spatial resolution of 250 x 250 m. EVI ranges from -1 (no vegetation) to 1 (complete vegetation).

Population density is used as a proxy for human influence on mosquito populations. Human activities provide both breeding grounds and dispersal opportunities (12). A higher population increases the probability of human-vector encounters thus increasing the risk of disease (2). Fine spatial resolution population density data was obtained from WorldPop. The WorldPop database provides population estimates from 2000–2023 and these population estimates are adjusted to match the United Nations estimates. The methodology for its curation has been described elsewhere (13) but in brief, it combines population data from national censuses and a suite of covariates via machine learning methods to create a gridded population surface at ~1 x 1 km spatial resolution (14,15).

Land cover is a representation of the physical material on the surface of the earth. The various land cover types affects *Aedes* species populations differently for instance croplands e.g. banana plantations can be important mosquito breeding areas as they retain the water required for breeding (16). Most *Aedes* species breed in tree holes which contain microbial food for its larvae (17). The fauna within forests can also provide blood sources for *Aedes* mosquitoes in the sylvatic cycle (18). While conversion of forests to built-up areas destroys their natural habitats, it may lead to increase in artificial environments such as artificial containers and tyres which also serve as breeding sites (19). The land cover layers were also obtained from MODIS, and they range from 2001–2023 at a spatial resolution of ~500 x 500m. Specifically, the International Geosphere-Biosphere Programme (IGBP) classification type was used and contains 17 classes. For Kenya, 14 of the 17 exists.

For the analyses, each class was represented on its own. To generate distinct covariate grid for each selected land cover variable, a reclassification was done: 1 to represent the actual class and 0 to represent the other classes. For instance, to produce a built-up raster surface, the original land cover raster was reclassified to two classes: 0 to represent the other classes and 1 to represent the built-up class. The process was repeated for the other land cover classes.

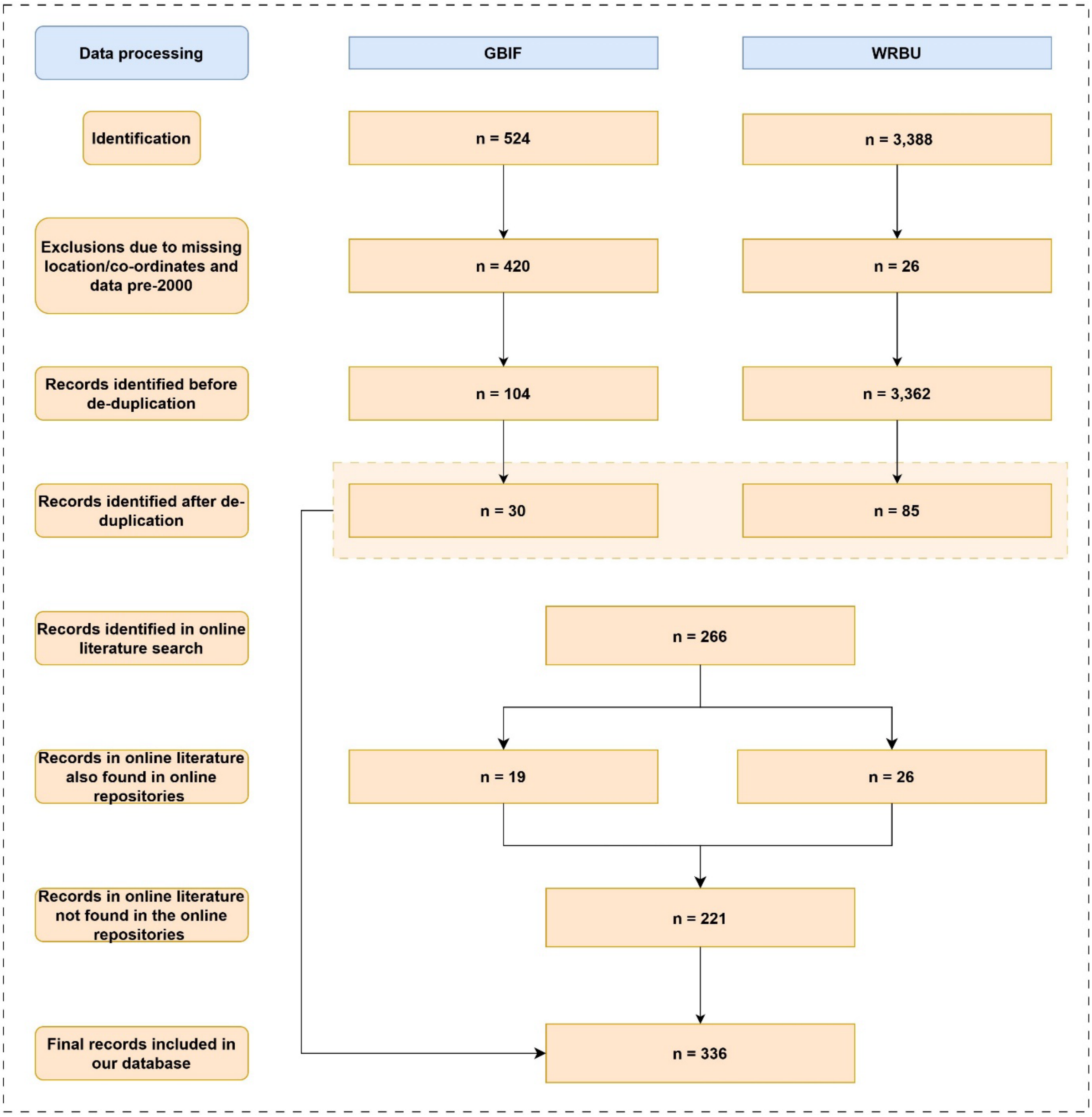

Figure SI 2: Workflow illustrating the acquisition and curation of *Aedes aegypti s.l.* occurrence data that were retrieved from online repositories — GBIF database (left) and WRBU VectorMap database (right) — and from online literature search.

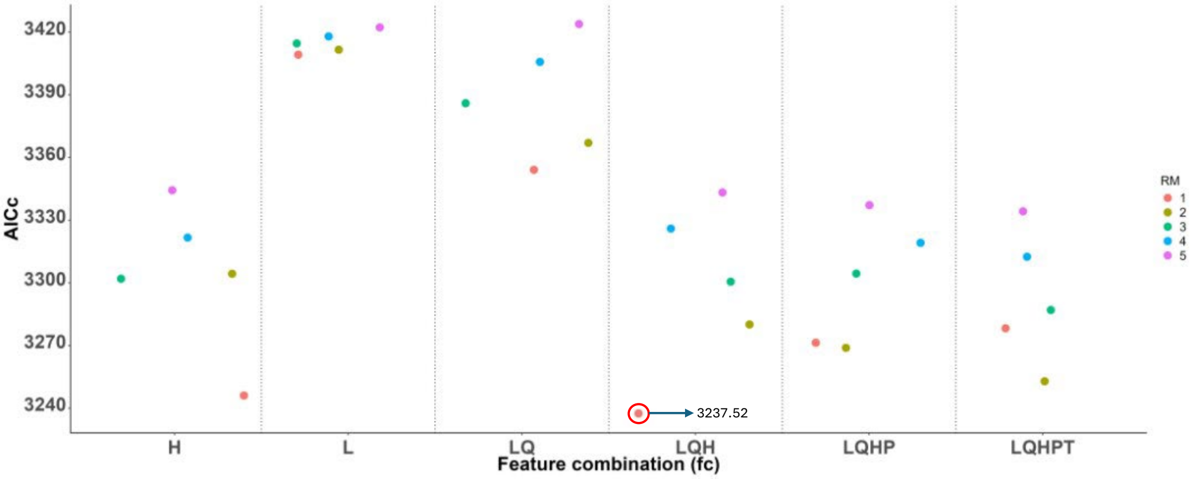

Figure SI 3: Corrected Akaike information criterion (AICc) for the preliminary MaxEnt models for *Ae. aegypti s.l.* using various feature combinations and regularisation multipliers.

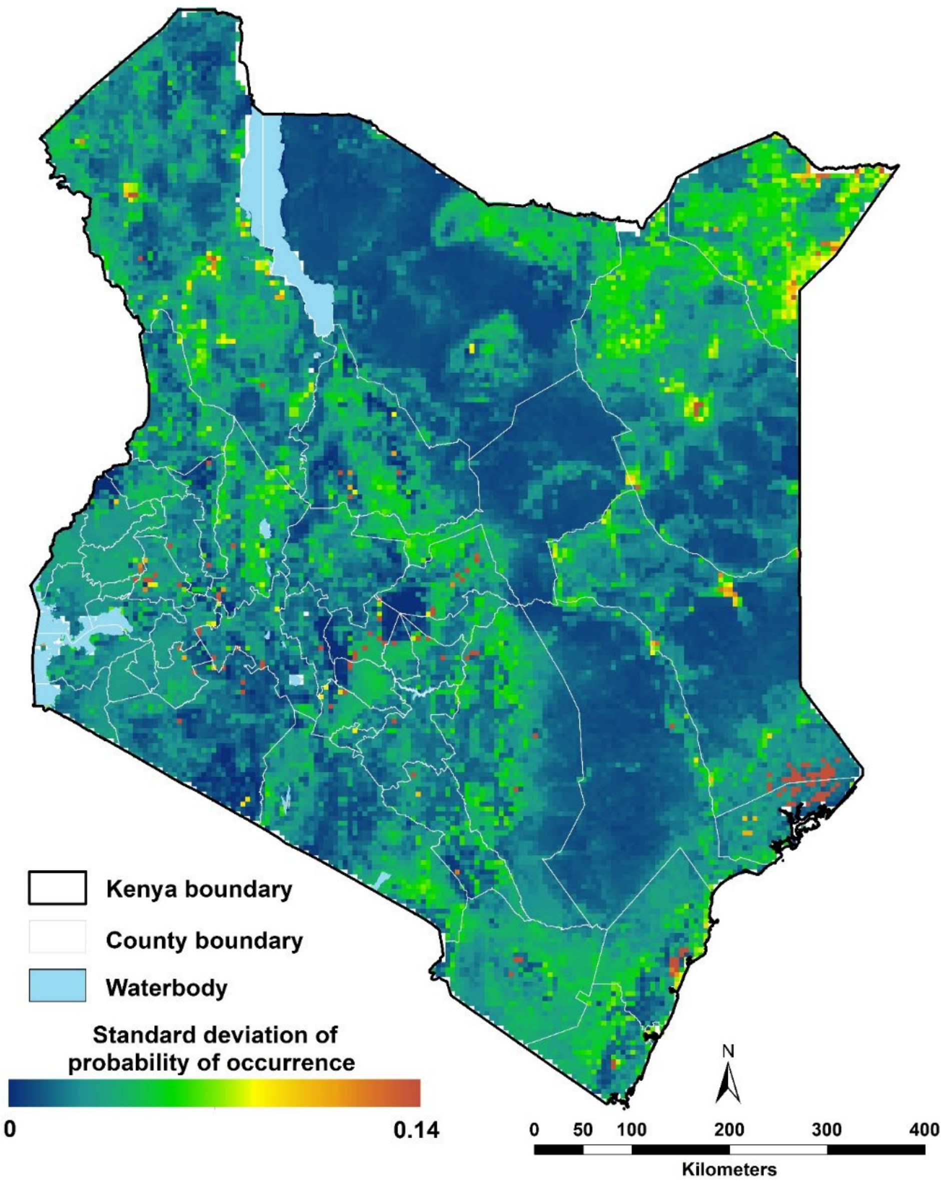

Figure SI 4: Map displaying the standard deviation associated with the probability of occurrence of *Ae. aegypti s.l.* predicted through our ecological niche modelling analyses.

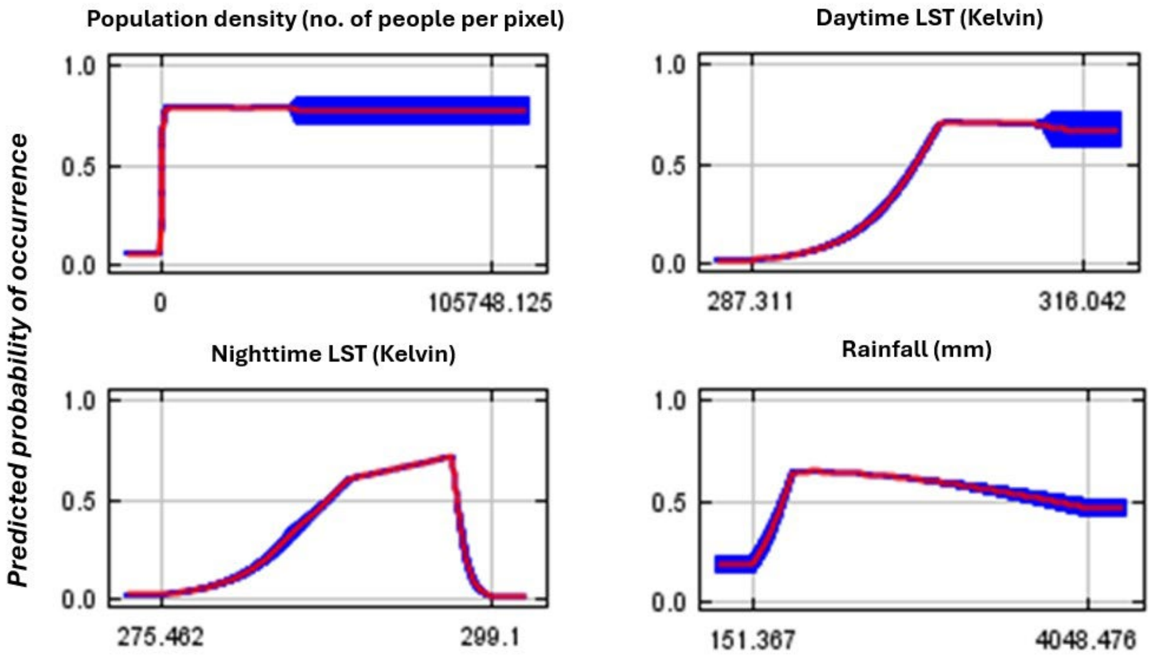

Figure SI 5: Response curves for environmental covariates included in our ecological niche modelling analyses.

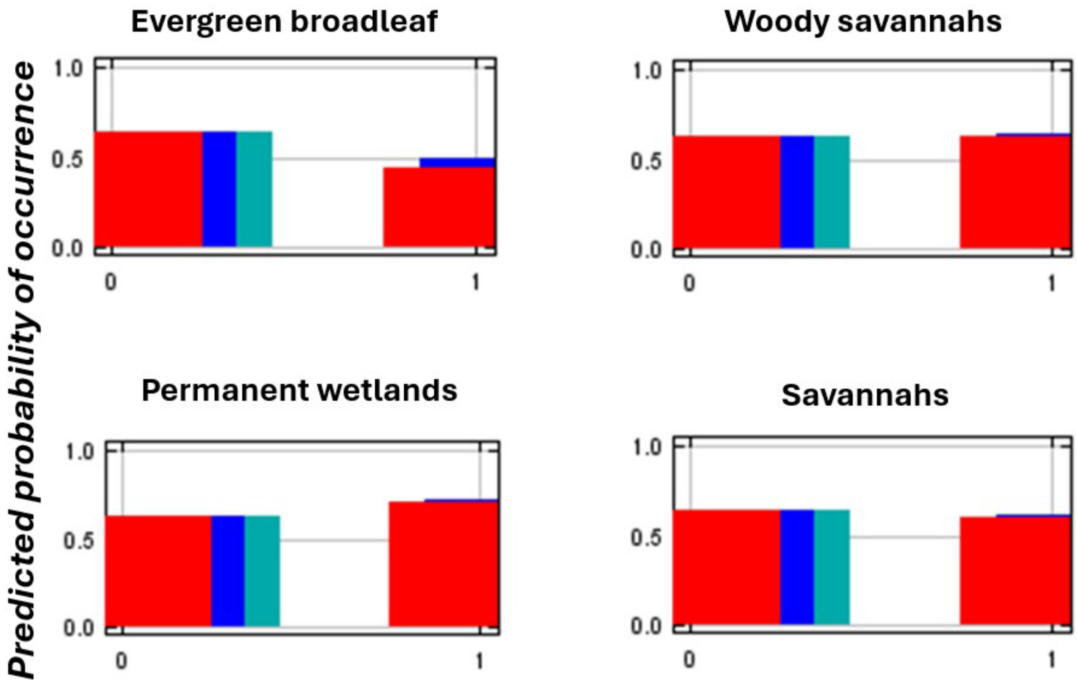

Figure SI 6: Response bar graphs for land cover variables used in our ecological niche modelling analyses.

References

1. Hafeez F, Naeem-Ullah U, Akram W, Arshad M, Iftikhar A, Naeem A, Saleem MJ. Habitat characterization of Aedes albopictus. *International Journal of Tropical Insect Science* (2022)1–6.
2. Padmanabha H, Durham D, Correa F, Diuk-Wasser M, Galvani A. The Interactive Roles of Aedes aegypti Super-Production and Human Density in Dengue Transmission. *PLoS Neglected Tropical Diseases* (2012) 6:1–11.
3. Romero-Vivas CM, Falconar AK. Investigation of relationships between Aedes aegypti egg, larvae, pupae, and adult density indices where their main breeding sites were located indoors. *Journal of the American Mosquito Control Association* (2005) 21:15–21.
4. Scott TW, Morrison AC, Lorenz LH, Clark GG, Strickman D, Kittayapong P, Zhou H, Edman JD. Longitudinal studies of Aedes aegypti (Diptera: Culicidae) in Thailand and Puerto Rico: population dynamics. *Journal of medical entomology* (2000) 37:77–88.
5. Agha SB, Chepkorir E, Mulwa F, Tigoi C, Arum S, Guarido MM, Ambala P, Chelangat B, Lutomiah J, Tchouassi DP. Vector competence of populations of Aedes aegypti from three distinct cities in Kenya for chikungunya virus. *PLoS Neglected Tropical Diseases* (2017) 11:e0005860.
6. Funk C, Verdin A, Michaelsen J, Peterson P, Pedreros D, Husak G. A global satellite-assisted precipitation climatology. *Earth System Science Data* (2015a) 7:275–287.
7. Funk C, Peterson P, Landsfeld M, Pedreros D, Verdin J, Shukla S, Husak G, Rowland J, Harrison L, Hoell A, et al. The climate hazards infrared precipitation with stations—a new environmental record for monitoring extremes. *Scientific Data* (2015b) 2:1–21.
8. Grimes DI, Pardo-Igúzquiza E. Geostatistical Analysis of Rainfall. *Geographical Analysis* (2010) 42:136–160.
9. Asgarian TS, Moosa-Kazemi SH, Sedaghat MM. Impact of meteorological parameters on mosquito population abundance and distribution in a former malaria endemic area, central Iran. *Heliyon* (2021) 7:e08477.
10. Estallo EL, Lamfri MA, Scavuzzo CM, Almeida FFL, Introini MV, Zaidenberg M, Almirón WR. Models for predicting Aedes aegypti larval indices based on satellite images and climatic variables. *Journal of the American Mosquito Control Association* (2008) 24:368–376.
11. Nihei N, Komagata O, Mochizuki K, Kobayashi M. Geospatial analysis of invasion of the Asian tiger mosquito Aedes albopictus: competition with Aedes japonicus japonicus in its northern limit area in Japan. *Geospatial Health* (2014) 8:417–427. doi: 10.4081/gh.2014.30
12. Khatchikian CE, Dennehy JJ, Vitek CJ, Livdahl TP. Environmental effects on bet hedging in Aedes mosquito egg hatch. *Evolutionary Ecology* (2010) 24:1159–1169.
13. Stevens FR, Gaughan AE, Linard C, Tatem AJ. Disaggregating census data for population mapping using random forests with remotely-sensed and ancillary data. *PLoS One* (2015) 10:e0107042. doi: 10.1371/journal.pone.0107042
14. Bondarenko M, Nieves J, Sorichetta A, Stevens FR, Gaughan AE, Tatem A. wpgpRFPMS: WorldPop random forests population modelling R scripts, version 0.1. 0. (2018)
15. Breiman L. Random forests. *Machine Learning* (2001) 45:5–32.
16. Diallo D, Sall AA, Buenemann M, Chen R, Faye O, Diagne CT, Faye O, Ba Y, Dia I, Watts D, et al. Landscape ecology of sylvatic chikungunya virus and mosquito vectors in southeastern Senegal. *PLoS Neglected Tropical Diseases* (2012) 6:e1649.

17. Vasilakis N, Cardosa J, Hanley KA, Holmes EC, Weaver SC. Fever from the forest: prospects for the continued emergence of sylvatic dengue virus and its impact on public health. *Nature Reviews Microbiology* (2011) 9:532–541. doi: 10.1038/nrmicro2595
18. Huang YS, Higgs S, Horne KM, Vanlandingham DL. Flavivirus-mosquito interactions. *Viruses* (2014) 6:4703–4730.
19. Zahouli JB, Utzinger J, Adja MA, Müller P, Malone D, Tano Y, Koudou BG. Oviposition ecology and species composition of Aedes spp. and Aedes aegypti dynamics in variously urbanized settings in arbovirus foci in southeastern Côte d'Ivoire. *Parasites & Vectors* (2016) 9:523. doi: 10.1186/s13071-016-1778-9
